# Management of advanced mycosis fungoides and Sézary Syndrome: International Recommendations for systemic treatments and allogeneic hematopoietic cell transplantation from the European Hematology Association, the EORTC Cutaneous Lymphoma Tumours Group, the International Society for Cutaneous Lymphoma, and the Lymphoma Working Party of the EBMT

**DOI:** 10.1002/hem3.70505

**Published:** 2026-11-05

**Authors:** Francesco Onida, Pietro Quaglino, Julia Scarisbrick, Anna Sureda, Emmilia Hodak, Evangelia Papadavid, Silvia Alberti‐Violetti, Martine Bagot, Lorenzo Cerroni, Amrita Goyal‐O'Leary, Rudolf Stadler, Rein Willemze, Jasmine Zain, Marco Ardigò, Emilio Berti, Adèle de Masson, Rafael F. Duarte, Francine Foss, Eva González‐Barca, Joan Guitart, Pablo Ortiz‐Romero, Alessandro Pileri, Pierluigi Porcu, Henry M. Prince, Sima Rozati, Serena Rupoli, Maarten H. Vermeer, Sean Whittaker, Nicola Pimpinelli, Pier Luigi Zinzani

**Affiliations:** ^1^ Hematology and BMT Unit, ASST Fatebenefratelli‐Sacco Milan Italy; ^2^ Department of Oncology and Hemato‐Oncology University of Milan Milan Italy; ^3^ Department of Medical Sciences Dermatologic Clinic, University of Turin Turin Italy; ^4^ Department of Dermatology University Hospitals Birmingham and Department of Immunology and Immunotherapy University of Birmingham UK; ^5^ Clinical Hematology Department Institut Català d'Oncologia‐Hospitalet, Institut d'Investigació Biomèdica de Bellvitge (IDIBELL), University of Barcelona Barcelona Spain; ^6^ Cutaneous Lymphoma Service, Davidoff Cancer Centre, Rabin Medical Center, Gray Faculty of Medicinal and Health Sciences, Tel Aviv University Tel Aviv Israel; ^7^ Department of Dermatology National and Kapodistrian University of Athens, National Centre for Rare Diseases‐Cutaneous Lymphoma, Attikon University Hospital Athens Greece; ^8^ Dermatology Unit, Fondazione IRCCS Ca' Granda Ospedale Maggiore Policlinico Milan Italy; ^9^ Department of Pathophysiology and Transplantation University of Milan Milan Italy; ^10^ Department of Dermatology Université Paris Cité Hospital St‐Louis Paris France; ^11^ Department of Dermatology Research Unit Dermatopathology, Medical University of Graz Graz Austria; ^12^ Department of Dermatology University of Minnesota Minneapolis Minnesota USA; ^13^ Department of Dermatology Johannes Wesling University Hospital, Ruhr University Bochum Minden Germany; ^14^ Department of Dermatology Leiden University Medical Center Leiden The Netherlands; ^15^ Lymphoma Service, Department of Medicine Memorial Sloan Kettering Cancer Center New York New York USA; ^16^ Dermatology Unit IRCCS Humanitas Research Hospital, Rozzano Milan Italy; ^17^ Department of Biomedical Sciences Humanitas University, Pieve Emanuele Milan Italy; ^18^ Inter‐Hospital Division of Pathology IRCCS MultiMedica Milan Italy; ^19^ Department of Dermatology Saint Louis Hospital, Université Paris Cité, Institut National de la Santé et de la Recherche Médicale (INSERM) U1342 Paris France; ^20^ Department of Hematology Hospital Universitario Puerta de Hierro Majadahonda, Universidad Autónoma de Madrid Madrid Spain; ^21^ Hematology and Stem Cell Transplantation, Yale University School of Medicine New Haven Connecticut USA; ^22^ Department of Dermatology Northwestern University Feinberg School of Medicine Chicago Illinois USA; ^23^ Department of Dermatology Institute i+12, Hospital 12 de Octubre, Medical School, University Complutense Madrid Spain; ^24^ Unit of Dermatology IRCCS University Hospital of Bologna Bologna Italy; ^25^ Department of Medical and Surgical Sciences Alma Mater Studiorum University of Bologna Bologna Italy; ^26^ Division of Hematology and BMT, Department of Internal Medicine Markey Cancer Center, University of Kentucky Lexington Kentucky USA; ^27^ Epworth Healthcare and Sir Peter MacCallum Department of Oncology The University of Melbourne Melbourne Victoria Australia; ^28^ Department of Dermatology Johns Hopkins University Baltimore Maryland USA; ^29^ Hematology Clinic, Azienda Ospedaliera Universitaria delle Marche Ancona Italy; ^30^ St John's Institute of Dermatology Guy's and St Thomas’ NHS Foundation Trust London UK; ^31^ Department of Health Sciences Section of Dermatology, University of Florence Florence Italy; ^32^ IRCCS Azienda Ospedaliero‐Universitaria di Bologna. Istituto di Ematologia “Seràgnoli”, Azienda Ospedaliero‐Universitaria di Bologna ‐ IRCCS Bologna Italy; ^33^ Dipartimento di Medicina Specialistica Diagnostica e Sperimentale, Università di Bologna Bologna Italy

## Abstract

Advanced mycosis fungoides (MF) and Sézary syndrome (SS) are the most aggressive forms of cutaneous T‐cell lymphoma and remain associated with a poor prognosis and heterogeneous therapeutic approaches. Recent advances, including targeted therapies and improved transplant strategies, have substantially expanded treatment options, highlighting the need for updated international guidance integrating dermatologic, hematologic, and transplant perspectives. An international multidisciplinary panel convened by the European Hematology Association (EHA), the EORTC Cutaneous Lymphoma Tumours Group (EORTC‐CLTG), the International Society for Cutaneous Lymphoma (ISCL), and the EBMT Lymphoma Working Party reviewed the available evidence and developed consensus recommendations for the management of advanced MF and SS. Thirty international experts participated in the process. Recommendations address diagnostic work‐up, systemic therapies, treatment sequencing, combination strategies, and indications for allogeneic hematopoietic cell transplantation (allo‐HCT). The consensus supports a stage‐adapted and compartment‐oriented therapeutic approach. Immunomodulatory strategies remain central in advanced CTCL: extracorporeal photopheresis is commonly used as first‐line therapy in SS and in patients with significant blood involvement, while interferon‐α and retinoids are widely used in MF, often in combination with skin‐directed therapies. Mogamulizumab and brentuximab vedotin represent key options after prior systemic therapy, with differential activity across skin, blood, and nodal compartments. Combination approaches integrating systemic and skin‐directed treatments may improve response depth and durability. Practical treatment algorithms for advanced MF and SS are proposed to support therapeutic sequencing and multidisciplinary management. Allo‐HCT remains the only potentially curative treatment and should be considered early in selected patients with high‐risk disease features.

## INCIDENCE AND EPIDEMIOLOGY, DIAGNOSTIC WORK‐UP, AND DISEASE STAGING

### Incidence and epidemiology

Mycosis fungoides (MF) and Sézary syndrome (SS) represent the most common forms of cutaneous T‐cell lymphomas (CTCL). While MF classically presents with patch and plaque skin lesions that may progress over time to tumors, erythroderma, and/or extracutaneous involvement, up to one‐third of MF patients may present with advanced‐stage disease at diagnosis. SS is typically characterized by generalized erythroderma and the leukemic presence of malignant T‐cells in the peripheral blood, with or without lymphadenopathy.

From an epidemiological perspective, MF/SS predominantly affects older adults, with a clear male predominance (M:F 2:1), and most commonly arises in the sixth decade of life.^1^ Although pediatric and adolescent cases are generally regarded as rare in Europe and in the United States, considerable geographic heterogeneity exists, with substantially different incidence rates reported worldwide.^2^ Nevertheless, available epidemiological data remain limited and are largely derived from national or regional cancer registries. In the United States, Surveillance, Epidemiology, and End Results (SEER) data report an annual incidence of approximately 4–6 cases per million inhabitants. Comparable estimates have been reported by European registries, including those from the Netherlands, Sweden, and France,^3^, ^4^, ^5^, ^6^ as well as from Australia.^7^

Population‐based studies consistently confirm the rarity of CTCL, with incidence rates generally below 1 per 100,000 person‐years. A modest increase in prevalence over time has been observed, likely reflecting improved diagnostic awareness and refined classification rather than a true increase in disease occurrence.

Importantly, prognosis remains poor in advanced‐stage disease. Data from the PROCLIPI study, a large international prospective observational cohort of patients with MF and SS, have demonstrated a median overall survival of less than 5 years in advanced stages.^1^, ^6^ In addition, racial and geographic disparities have been described, with Black patients often presenting at a younger age and with more advanced disease at diagnosis compared with other populations.^3^, ^8^, ^9^

### Diagnostic work‐up

The diagnostic approach differs significantly between MF and SS, and varies according to disease stage and presentation. Tables 1, 2, 3, 4 summarize the current consensus on diagnosis, staging, and re‐evaluation across different clinical scenarios.

**Table 1:** Diagnosis and staging work‐up for MF/SS.

	Patients presenting with patches and plaques (T1/T2)	Patients presenting with tumors (T3)	Patients presenting with erythroderma (T4)	Additional notes
**DIAGNOSIS**				
**SKIN BIOPSY**				
**Biopsy site selection and technique**	Punch biopsies of ≥5 mm are recommended. Multiple biopsies may be necessary to capture the pathologic variability of disease at diagnosis. In the setting of early‐stage disease, biopsies may be nondiagnostic, and multiple serial biopsies may be necessary to make a diagnosis. If multiple morphologies are present, each should be sampled. If only a single sample can be taken, the most indurated area should be biopsied. If a single biopsy is diagnostic, an additional biopsy is not necessary. In the case of a nondiagnostic biopsy, but ongoing clinical suspicion, repeat biopsy of a lesion that has been untreated for 4 weeks may be helpful.	Multiple biopsies are recommended: biopsy of both an earlier (patch/plaque) lesion and the most prominent/progressive lesions is essential to differentiate tumor stage MF from other aggressive forms of CTCL and other T‐cell lymphomas (C‐ALCL; PTCL, NOS)	Biopsy of erythrodermic skin that is not eroded or secondarily infected. Histology may be nonspecific. Two ≥5 mm punch biopsies or a single elliptical biopsy may be sufficient. If patches, plaques, or tumors are also present, each morphology should also be sampled.	Nonspecific skin biopsy findings in the setting of suspected Sézary syndrome may support the exclusion of other nonmalignant causes of erythroderma (e.g., atopic dermatitis and drug eruption); however, in a proportion of SS cases, histopathologic findings may also appear as nonspecific dermatitis, underscoring the need for clinicopathologic correlation and peripheral blood evaluation. Demonstration of an identical T‐cell clone in skin and peripheral blood may further support the diagnosis in challenging cases.
**Immunohistochemical markers for evaluation of tissue biopsy**	Necessary: CD2, CD3, CD4, CD5, CD7, CD8, and CD20. Potentially helpful: CD1a, TCRB, TCRD, TIA‐1, granzyme B, perforin, PD‐1, CXCL13, Ki‐67/MIB‐1, CD68, CD163, and CD30.	Necessary: CD2, CD3, CD4, CD5, CD7, CD8, CD20, **CD30, CD56,** and **EBER.** Potentially helpful: TCRB, TCRD, TIA‐1, granzyme B, perforin, PD‐1, ICOS, and Ki‐67/MIB‐1.	Necessary: CD2, CD3, CD4, CD5, CD7, CD8, CD20, CD30, and **PD‐1.** Potentially helpful: CCR4, PD‐1, TOX, and Ki‐67/MIB‐1.	All cases. A dermatopathologist with expertise in the diagnosis of CTCLs is strongly recommended.
				Patch/Plaque disease − **CD1a** can help differentiate CD4+ lymphocytes and Langerhans cells in the epidermis, particularly when distinguishing MF from lymphomatoid contact allergic dermatitis. − **CD68 and CD163** can aid in interpretation of CD4+ cells in the dermis. − **CD30** is indicated in cases where lymphomatoid papulosis, anaplastic lymphoma, or large‐cell transformation is considered.
				Tumors − the **CD56 and EBER** may be useful in diagnosis of advanced‐stage disease to rule out other CTCLs. − **PD‐1 and ICOS** are particularly important in the setting of solitary lesions to rule out primary cutaneous CD4^+^ small/medium T‐cell lymphoproliferative disorder (PCSM‐TCL).
				Erythroderma − **CCR4** may be helpful in cases where mogamulizumab is used. − **PD‐1** positivity >90% is highly supportive of a diagnosis of Sezary syndrome versus a nonmalignant/reactive/inflammatory dermatosis.
**EVALUATION FOR CLONAL GENE REARRANGEMENTS OF** ***TCRB*** **AND** ***TCRG***				
**Skin/tissue biopsy**	May be a helpful diagnostic adjunct in the appropriate clinical setting.	Required.	Required.	In early‐stage disease, up to 50% of samples may be negative. Clonal TCR gene rearrangements are *supportive* of a diagnosis of lymphoma but *not sufficient*. Clonal TCR gene rearrangements can also be seen in patients with nonmalignant conditions. Results should be interpreted in the context of the overall presentation. Assessment of TCR clonality is necessary in the setting of advanced disease, progression, or Sezary syndrome to confirm the clonal relation of multiple lesions.
**Blood**	Optional, but ideally obtained in all cases to permit accurate staging of blood involvement.	Required.	Required.	Blood TCR evaluation is mandatory in the setting of suspected blood involvement, advanced‐stage disease, suspected progression, or erythroderma. Standardized flow cytometry approaches, including EUROFLOW‐based panels when available, are encouraged to improve reproducibility and inter‐laboratory comparability. Any clonal TCR gene rearrangement in the blood must be compared with and found to be identical to that in the skin.
**STAGING**				
**EVALUATION FOR CIRCULATING BLOOD INVOLVEMENT**				
**Quantification of circulating Sezary cells via flow cytometry**	Optional.	Required.	Required.	Flow cytometry should be performed in the setting of suspected adenopathy, rapid progression, or suspected extracutaneous disease to identify correct B stage. The diagnosis of Sezary syndrome or erythrodermic MF requires quantification of circulating neoplastic cells. Flow cytometry panel should minimally include CD3, CD4, CD7, CD8, CD26, and CD45. TRBC1 may be helpful when available. To calculate the number of circulating atypical cells from flow cytometry data: Absolute Sézary count = Total lymphocyte count x percent of the atypical T‐cell population.
**Peripheral smear**	Optional.	Optional.	Required in SS to exclude other mature hematologic malignancies (ATLL, TPLL).	Sezary cell count is typically no longer performed. It is less useful than flow cytometry due to the subjective nature of the process but may still be useful in cases where an aberrant immunophenotype is not detected.
**EVALUATION FOR LYMPH NODE AND VISCERAL DISEASE**				
**Imaging with PET‐CT scan**	Optional for T1 (BSA < 10%). Consider for T2a (patch disease with ≥10% BSA). Necessary for T2b (patch/plaque disease with ≥10% BSA).	Necessary.	Necessary.	Imaging is necessary for ≥T2b, LCT, FMF, palpable adenopathy, or abnormal laboratory studies (e.g., elevated LDH or β2‐microglobulin, unexplained lymphocytosis, or abnormal peripheral blood immunophenotype suggesting blood involvement). Imaging may also be considered in patients with extensive plaque disease (> 10% body surface area). PET‐CT strongly preferred, but if not available, CT scans of the chest, abdomen, pelvis, and neck may be performed. Choice of imaging modality may also consider cumulative radiation exposure and local availability, as radiation dose may vary depending on the specific PET‐CT or CT technology used.
**Excisional lymph node biopsy**	Necessary in patients with a node that is either ≥1.5 cm in diameter and/or is firm, irregular, clustered, or fixed.	Necessary in patients with a node that is either ≥1.5 cm in diameter and/or is firm, irregular, clustered, or fixed.	Necessary in patients with a node that is either ≥1.5 cm in diameter and/or is firm, irregular, clustered, or fixed.	If a PET‐CT scan is performed, biopsy of the node with the highest FDG uptake is recommended. Excisional or adequate core needle biopsy is necessary. **A fine‐needle aspiration (FNA) biopsy alone is insufficient** for the initial diagnosis of lymphoma. Re‐biopsy if pathological findings are nondiagnostic and/or discordant with the clinical presentation. Pathologic evaluation of a positive node requires grading according to either the Dutch Criteria for Lymph Nodes in CTCL or the NCI‐VA Lymph Node Classification for MF.
**Bone marrow biopsy**	Not recommended for routine diagnostic purposes.	Not recommended for routine diagnostic purposes.	Not recommended for routine diagnostic purposes.	Data regarding the significance of bone marrow involvement in MF/SS are limited. Bone marrow biopsy should be performed only when required for other indications (e.g., unexplained hematologic abnormalities). Bone marrow involvement is considered visceral involvement, and is defined as a nodular, diffuse, or interstitial involvement (>5% of BM cellularity) with immunophenotype and clonal TCR consistent with the skin.
**LABORATORY STUDIES**				
**General laboratory assessments**	CBC with manual differential, liver function tests, LDH, and comprehensive chemistries.	CBC with manual differential, liver function tests, LDH, and comprehensive chemistries.	CBC with manual differential, liver function tests, LDH, and comprehensive chemistries.	LDH elevation is independently associated with poorer prognosis. B2‐microglobulin can be considered for T3/T4 disease. Beta‐HCG to evaluate for pregnancy should be performed in the appropriate setting and based on the proposed treatment.
**HTLV‐1 serologic testing**	Not routinely performed in early‐stage MF but may be helpful in challenging cases in the appropriate patient population.	Recommended in patients from endemic/high‐prevalence areas.	Recommended in patients from endemic/high‐prevalence areas.	HTLV‐1 is known to be endemic to the south‐western part of Japan; some parts of the Caribbean and surroundings regions; foci in South America; some areas of intertropical Africa, the Middle East, and Romania; and rare isolated clusters in Australia and Melanesia. HTLV‐1 prevalence is not homogeneous even in endemic areas and has not been investigated in large regions of the world. https://www.ecdc.europa.eu/sites/default/files/media/en/publications/Publications/geographical-distribution-areas-high-prevalence-HTLV1.pdf

**Table 2:** Re‐staging requires assessment of each disease compartment: skin (T), lymph node (N), viscera (M), and blood (B).

RESTAGING OF SKIN (T), LYMPH NODE (N), VISCERA (M), AND BLOOD (B).	
**SKIN**	
Full body skin exam with calculation of BSA	Calculation of the mSWAT score is highly recommended.
Repeat biopsy of skin lesions (if indicated)	See Table 3 for possible indications for re‐biopsy. Multiple biopsies may be needed and should be performed in different anatomic areas. In the setting of a clear diagnosis of MF, a biopsy of the area suspicious for progression or large‐cell transformation may be sufficient. This is typically a more infiltrated area. If LCT has previously been histologically confirmed, additional biopsy may not be necessary, particularly when the primary concern is nodal, blood, or visceral progression.
Tissue TCR clonality (required if repeat biopsy performed)	See Table 1 for more details.
**LYMPH NODE/VISCERA**	
Imaging	PET‐CT is strongly preferred, but if not available, CT scans of the chest, abdomen, pelvis, and neck may be performed.
Excisional or adequate core needle lymph node biopsy (if indicated and lymph node involvement has not been previously pathologically confirmed)	Necessary in patients with a node that is either ≥1.5 cm in diameter and/or is firm, irregular, clustered, or fixed. If a PET‐CT scan is performed, biopsy of the node with the highest FDG uptake is recommended. Excisional or adequate core needle biopsy is necessary. **A fine‐needle aspiration (FNA) biopsy alone is insufficient** for the initial diagnosis of lymphoma. Pathology evaluation of a positive node requires grading according to either *the Dutch Criteria for Lymph Nodes* in CTCL or the *NCI‐VA Lymph Node Classification* for MF.
**BLOOD**	
Peripheral blood flow cytometry for circulating Sezary cells	See Table 1 for more details.
Peripheral blood TCR clonality	See Table 1 for more details

**Table 3:** Examples of indications for re‐biopsy of skin lesions.

EXAMPLE INDICATIONS FOR RE‐BIOPSY OF SKIN LESIONS	
Suspected disease progression (and large‐cell transformation not previously pathologically confirmed)	*Potential signs of progression may include, but are not limited to*: − Changes in lesion morphology. − Evolution from patches/plaques to tumors. − New erythroderma. − New involvement of unusual body areas (mucosa and palms/soles).
Suspicion for new large‐cell transformation (and not previously pathologically confirmed)	*Potential signs of LCT may include, but are not limited to:* − New tumors. − Marked increase in plaque thickness or nodularity.
Suspicion for new folliculotropic MF (and not previously pathologically confirmed)	*Potential signs of new folliculotropic involvement may include, but are not limited to:* − New folliculotropism on histopathology. − New head or neck involvement. − Appearance of follicular papules. − New‐onset alopecia in affected areas.
Exclusion of other possible conditions such as drug rash	For example, mogamulizumab‐associated rash and drug hypersensitivity rashes. Comparison of pre‐ and posttreatment biopsies may aid in distinguishing MAR from disease progression.
Lack of response to treatment	Particularly when a lesion fails to respond to treatment while others improve, and when this cannot be explained by treatment‐related factors (e.g., sanctuary sites or limited exposure to skin‐directed therapy), to evaluate for large‐cell transformation or alternative pathology.
Initial biopsy non‐diagnostic/other diagnostic uncertainty	

**Table 4:** Examples of indications for re‐staging work‐up.

EXAMPLES OF INDICATIONS FOR RESTAGING WORK‐UP
Marked increase in body surface area involvement or new onset of tumoral lesions
Histologic evidence of new large‐cell transformation
Histologic evidence of new folliculotropic involvement suggestive of progression from early‐ to advanced‐stage disease*
New erythroderma
New lymphadenopathy
Constitutional symptoms (fever, night sweats, fatigue, and weight loss)
Lack of response to treatment
Aggressive clinical behavior
Based on physician clinical judgment

Diagnosis requires integration of clinical, histopathologic, immunophenotypic, and molecular findings. Skin biopsy remains a cornerstone of the diagnostic work‐up. In particular, multiple punch biopsies obtained from different anatomical sites and, when necessary, at different time points are recommended, especially in early‐stage disease, where histopathologic features may be subtle or non‐specific. While 4‐mm punch biopsies are commonly used in routine clinical practice, larger punch biopsies (ideally 5–6 mm when feasible) may increase diagnostic yield by providing a broader and deeper tissue sample, allowing improved assessment of epidermotropism and adnexal involvement, and reducing sampling variability. Immunohistochemical assessment should include both mandatory and adjunctive markers, as detailed in Table 1. T‐cell receptor (TCR) clonality analysis on skin specimens is recommended in all advanced‐stage cases and may also be informative in early‐stage disease. Whenever possible, molecular studies should be performed on adequately sized tissue samples to ensure sufficient DNA yield; comparison with blood clonality aids in the identification of systemic involvement. All histopathological specimens should be reviewed by a dermatopathologist with specific expertise in CTCL.

### Staging at diagnosis

Staging follows the TNMB system (Tables 5 and 6).

- Skin (T): body surface area and the mSWAT score; clinical photo documentation may be useful, when available, to document skin involvement over time.
- Nodes (N): clinical and imaging evaluation (PET‐CT preferred), with biopsy performed when clinically indicated, particularly in enlarged (>1.5 cm) or PET‐avid nodes.
- Viscera (M): imaging evaluation, with biopsy confirmation recommended for suspected visceral involvement whenever feasible, except for liver and spleen involvement, which may be diagnosed radiologically.^10^
- Blood (B): flow cytometry^11^ and TCR clonality by PCR or NGS techniques.

**Table 5:** TNMB staging of MF/SS.

TNMB staging of MF/SS					
**Skin (T)**	**T1**	Patches, plaques, or papules <10% BSA	**T1A**	Patch/papule only	**Patch** = Any size skin lesion without significant elevation or induration. **Plaque** = Any size skin lesion that is elevated or indurated. **Papule** = Small, circumscribed, elevated solid skin lesion, typically <1 cm in diameter. **Tumor** = at least one ≥1 cm diameter solid or nodular lesion with evidence of depth and/or vertical growth.
			**T1B**	Plaque ± patch	
	**T2**	Patches, plaques, or papules, ≥10% BSA	**T2A**	Patch/papule only	
			**T2B**	Plaque ± patch	
	**T3**	One or more tumors ≥1 cm in diameter			
	**T4**	Confluence of erythema covering ≥80% BSA			
**Node (N)**	**N0**	No clinically abnormal LN (no biopsy necessary)			**NCI‐VA Lymph Node Classification** LN0: no atypical lymphocytes. LN1: occasional and isolated atypical lymphocytes. LN2: many atypical lymphocytes or in 3–6 cell clusters. LN3: aggregates of atypical lymphocytes; nodal architecture preserved. LN4: partial/complete effacement of nodal architecture. **Dutch Criteria for Lymph Nodes** Grade 1: Dermatopathic lymphadenopathy. Grade 2: Early involvement by mycosis fungoides. Grade 3: Partial effacement of lymph node architecture. Grade 4: Complete effacement of lymph node architecture.
	**N1**	Dutch grade 1 or NCI LN 0‐2			
	**N2**	Dutch grade 2 or NCI LN3			
	**N3**	Dutch grade 3–4 or NCI LN4			
	**Nx**	Clinically abnormal node but no pathologic determination of representative LN			
**Visceral (M)**	**M0**	No visceral involvement			**Bone marrow** involvement is considered visceral involvement, and is defined as a nodular, diffuse, or interstitial involvement (>5% of BM cellularity) with immunophenotype and clonal TCR consistent with the skin.
	**M1**	Visceral involvement	**M1a**	BM‐only involvement	
			**M1b**	Non‐BM visceral involvement	
	**Mx**	Visceral involvement is neither confirmed nor refuted			
**Blood (B)**	**B0**	Absence of significant blood involvement			**B0** = ≤250/µL of CD4+/CD26− or CD4+/CD7−. **B1** = does not meet criteria for B0 or B2. **B2** = ≥1000/µL of CD4+/CD26− or CD4+/CD7− cells. Note: In patients with marked lymphopenia, absolute counts of aberrant T‐cells may underestimate blood involvement and should be interpreted in the context of overall lymphocyte counts and flow cytometry results.
	**B1**	Low blood tumor burden			
	**B2**	High blood tumor burden			
	**Bx**	Unable to quantify blood involvement			

**Table 6:** Clinical staging for MF/SS.

Clinical stage		T	N	M	B
**IA**	**Limited skin involvement**	T1	N0	M0	B0‐1
**IB**	**Skin‐only disease**	T2	N0	M0	B0‐1
**IIA**		T1‐2	N1−2	M0	B0‐1
**IIB**	**Tumor stage disease**	T3	N0‐2	M0	B0‐1
**IIIA**	**Erythrodermic disease**	T4	N0‐2	M0	B0
**IIIB**		T4	N0‐2	M0	B1
**IVA1**	**Blood involvement** *	T1‐4	N0‐2	M0	B2
**IVA2**	**Nodal disease** *	T1‐4	N3	M0	B0‐2
**IVB**	**Visceral disease** *	T1‐4	N0‐3	M1	B0‐2

* Sézary syndrome is defined by erythroderma (T4), B2 blood involvement, and demonstration of an identical T‐cell clone in skin and peripheral blood. Although most patients with Sézary syndrome are classified as stage IVA1 or higher, stage IVA1 also includes non‐erythrodermic patients with B2 blood involvement.

Bone marrow biopsy is not routinely required and should be reserved for unexplained hematologic abnormalities.

### Re‐staging and diagnostic reassessment

Re‐staging is indicated in patients with advanced‐stage disease at progression, relapse, or transformation, and in early‐stage patients with suspected progression to advanced disease. Repeat biopsies are warranted when new lesions arise, transformation to large‐cell lymphoma is suspected, or treatment resistance occurs (Table 3).

### Prognosis

Prognosis in MF and SS is primarily related to clinical stage. While patients with early‐stage MF (IA–IIA) have excellent survival outcomes, those with advanced‐stage MF (IIB–IV) or SS have a significantly worse prognosis. The median overall survival in advanced‐stage patients is ~5 years in stages IIB‐IVA1, decreasing to <3 years in those with lymphomatous nodes (IVA2) or visceral disease (IVB).^1^, ^8^ A cutaneous lymphoma prognostic index “CLIPI” has recently been published that better stratifies patients with all stages of advanced MF/SS for overall survival according to the number of risk factors (serum LDH above ULN, age of diagnosis >60 years, LCT in skin, and N3 nodal involvement [defined as lymph nodes with complete architectural effacement; see Table 5), where low risk involves 0–1 factors (5‐year survival 63.3%), intermediate risk involves 2 factors (5‐year survival 44.7%), and high risk involves 3–4 factors (5‐year survival just 18.3%).^1^ This may allow better management planning.

## FIRST‐LINE SYSTEMIC TREATMENT OF ADVANCED‐STAGE MF

First‐line therapy for advanced‐stage MF (stages IIB–IV) should be individualized according to disease burden, comorbidities, treatment accessibility, and long‐term goals, including the feasibility of allogeneic hematopoietic cell transplantation (allo‐HCT). In transplant candidates, the objective is to achieve maximal disease control before HCT; in others, the objective is to reduce symptoms and tumor burden with minimal toxicity and preserve quality of life. Given the absence of prospective comparative trials and variability in drug availability, there is no universal standard of care, and treatment is primarily aimed at long‐term disease control, symptom reduction, and preservation of quality of life.

### Skin‐directed therapies (SDTs)

Phototherapy (PUVA or narrow‐band UVB), topical corticosteroids, topical calcineurin inhibitors (e.g., pimecrolimus), and topical chlormethine gel retain a supportive role in advanced MF, particularly for symptomatic lesions. Phototherapy may induce responses in erythrodermic MF (stage III), though relapses are frequent. SDT are frequently combined with systemic therapies to enhance local control.^12^

### Radiotherapy (RT)

Localized RT provides excellent local control in stage IIB disease with solitary or clustered nodules or plaques, often combined with systemic agents.^13^ Treatment is usually 8–12 Gy in two or three fractions. A high response rate was observed following low‐dose RT, with a 1‐year local control rate of ≥92%.^13^ Total skin electron beam therapy (TSEBI) is highly effective for generalized tumors or erythrodermic MF (stage IIIA), with overall response rates (ORRs) approaching 99%.^14^, ^15^ Low‐dose (8–12 Gy) or intermediate‐dose (20–24 GY) TSEBI is preferred over standard dose (30–36 Gy) for its tolerability and potential for retreatment.^13^ In retrospective series, the addition of ECP to TSEBI has been associated with improved disease control and survival outcomes in erythrodermic MF; however, these findings derive from nonrandomized data and should be interpreted with caution.^16^ Because responses are time‐limited, TSEBI should be integrated into a broader therapeutic plan, especially when allo‐HCT is anticipated.^15^

### Retinoids

Bexarotene, a rexinoid approved by both the FDA and the EMA, is indicated for MF refractory to prior therapy. The standard labeled dose is 300 mg/m^2^/day, alone or combined with SDT, extracorporeal photopheresis (ECP), or interferon‐α (IFN‐α).^17^, ^18^, ^19^ Lower doses (150–225 mg/m^2^/day) may retain efficacy with improved tolerability (ORR 37%–67%). Monitoring is required for teratogenicity, central hypothyroidism, and hyperlipidemia; thyroid replacement and lipid‐lowering therapy (preferably fenofibrate) are often necessary.^19^

Other retinoids, including acitretin and isotretinoin, are used off‐label. Acitretin has demonstrated moderate activity in early and folliculotropic MF, and may be combined with phototherapy or IFN‐α.^20^, ^21^, ^22^ It represents an option where bexarotene is unavailable or poorly tolerated.

### IFN‐α

After withdrawal of recombinant IFN‐α2a and ‐α2b, pegylated IFN‐α2a (45–180 mcg per week) remains the only available formulation (see Table 7). It may be used alone or in combination with SDT or bexarotene or MTX. In advanced MF, response rates are higher when combined therapy is used.^23^ Peg‐IFN‐α2a is generally well tolerated; transient flu‐like symptoms, fatigue, and hepatotoxicity are the most common adverse events, manageable by gradual dose escalation. Recently, due to a worldwide shortage of pegIFN‐α2a, a ropeginterferon‐α2b has emerged as a possible therapeutic alternative.^24^

**Table 7:** Dosages and principal adverse events of systemic therapies used in advanced MF.

Therapy	Dosage	Principal AE (>10%)	RARER AE (<10%)	References
Acitretin^a^	10–50 mg/day	Dyslipidemia, xerosis, cheilitis, alopecia, fragile nails, photosensitivity, liver function abnormalities, and teratogenicity.	Pancreatitis, hepatitis, anemia, leukopenia, or thrombocytopenia, and calcification of tendons or ligaments.	[20, 21, 22]
Alemtuzumab	10–30 mg (iv/sc) 3 times/week up to 12 weeks	Infusion‐associated reaction, hematologic toxicity, and mild to moderate infections (like CMV reactivation), secondary autoimmune disorders, and prolonged and profound lymphocyte depletion with associated immunosuppression.	Immune‐mediated pneumonitis, severe respiratory events, kounis syndrome, and myocarditis.	[25, 26, 27]
Bexarotene	300 mg/m^2^/day orally; can be started from 150–225 mg/m^2^/day to reduce side effects	Teratogenicity, hypertriglyceridemia, hypercholesterolemia, hypothyroidism, fatigue, nausea, headache, neutropenia, hypertransaminasemia, dryness of skin and mucous and photosensitivity, peripheral edema, weight loss, and diarrhea.	Pancreatitis, thrombocytopenia, anemia, hypocalcemia, visual disturbances, renal dysfunction, and major depression (occasionally severe and requiring treatment discontinuation).	[17, 18, 19]
Brentuximab vedotin	1.8 mg/kg intravenously every 3 weeks; dose reduction to 1.2 mg/kg or to 0.8 mg/kg may be required for those with worsening neuropathy.	Peripheral neuropathy, neutropenia, fatigue, nausea, infections, diarrhea, vomiting, alopecia, and fever.	Hepatotoxicity, cardiotoxicity, pulmonary toxicity, febrile neutropenia, insulin resistance, and Brentuximab‐associated rash (BVAR).	[28, 29, 30, 31, 32]
Extracorporeal Photopheresis	2 consecutive days every 2–4 weeks (conventional schedule); alternative 1‐day regimen: weekly for 6 weeks, then every 2 weeks, and then monthly.	Hypotension, anemia, and infection risk.		[33, 34, 35, 36, 37, 38, 39]
Gemcitabine	1000–1200 mg/m^2^ IV Days 1, 8, 15 every 28 days (various schedules reported).	Myelosuppression (neutropenia, thrombocytopenia, and anemia), fatigue, nausea, elevated transaminases, and flu‐like symptoms.	Severe infections, pneumonitis/interstitial lung disease, hemolytic–uremic syndrome, and severe cutaneous reactions.	[40, 41]
Histone Deacetylase Inhibitors (Vorinostat, Romidepsin)	Vorinostat: 400 mg/day; Romidepsin: 14 mg/m^2^ IV on Days 1, 8, and 15 of a 28‐day cycle.	Fatigue, nausea, thrombocytopenia, and QT prolongation		[42, 43, 44, 45, 46, 47]
Low‐dose methotrexate	5–35 mg/week orally or intramuscular or subcutaneously.	Nausea and vomiting, fatigue, stomatitis, hepatotoxicity, and cytopenia	Interstitial lung disease and severe myelosuppression.	[48, 49]
Mogamulizumab	1.0 mg/kg intravenously weekly for 5 weeks, and then every 2 weeks	Infusion reactions, MAR, nausea, chills, and headache.	Autoimmune cutaneous and systemic complications (e.g., alopecia areata, vitiligo, psoriasis, hepatitis, colitis, thyroiditis, glomerulonephritis, nephrotic syndrome, myasthenia gravis, myositis, and myocarditis).	[50, 51, 52, 53]
Peg‐IFN‐α2a	45–180 µg/week subcutaneously, or up to 180–360 µg/week in dose‐escalation studies.	Fatigue, flu‐like symptoms, neutropenia, elevated liver enzymes, anemia, and depression.	Autoimmune hepatitis, lupus‐like syndrome, cardiac arrhythmias, thyroid dysfunction, neuropathy, and acute kidney injury.	[23]
Pegylated liposomal doxorubicin	20–40 mg/m^2^ IV every 2–4 weeks	Myelosuppression, mucositis/stomatitis, fatigue, nausea, and diarrhea.	Cardiotoxicity, infusion‐related reactions, secondary infections, and secondary malignancies.	[54]
Pralatrexate	15 mg/m^2^ IV weekly for 3 of every 4 weeks.	Mucositis, fatigue, nausea, and cytopenias.	Hepatotoxicity, severe stomatitis, and infections.	[55, 56]

*Note*: Therapies are presented in alphabetical order and do not reflect a hierarchy of efficacy or clinical preference. Disclaimer: The adverse events listed in this table are not exhaustive. Clinicians should refer to the full prescribing information and local regulatory guidance for comprehensive safety data. Dose modifications may be required based on baseline comorbidities (e.g., hepatic or renal impairment, diabetes, and hyperlipidemia), concomitant medications, and patient‐specific factors. Special caution is warranted in vulnerable populations, including pregnant women and those at high risk of infections.

Abbreviations: AE, adverse events; MAR, mogamulizumab‐associated rash; Peg‐IFN, pegylated interferon α.

^a^ Acitretin is not specifically approved for MF/SS and may be used off‐label according to local practice and regulatory policies.

### ECP

ECP is an immunomodulatory therapy particularly effective in erythrodermic (stage III) and leukemic (stage IVA1) MF/SS, with ORR between 54% and 77%.^33^, ^34^, ^35^ It is characterized by a favorable safety profile, with minimal immunosuppression and no documented increase in secondary malignancies. The conventional schedule consists of treatment on two consecutive days every 2–4 weeks.^36^ Alternative regimens have also been described. In a large single‐center retrospective series, early use of a modified 1‐day ECP schedule (weekly for 6 weeks, then every 2 weeks, and then monthly) was associated with prolonged time to next treatment (TTNT) and encouraging overall survival in patients with SS and erythrodermic MF.^37^ A multicenter retrospective study in SS showed improved TTNT in patients receiving ECP‐containing regimens compared with non‐ECP approaches, particularly when ECP was introduced earlier in the treatment course.^38^ In multivariable analysis, ECP use was also associated with improved overall survival.^39^ These findings, although derived from nonrandomized data, support the early integration of ECP within the therapeutic algorithm. In clinical practice, ECP is typically continued until disease progression, relapse, or unacceptable toxicity, given its favorable tolerability and suitability for long‐term administration. Combination with methotrexate (MTX), bexarotene, or interferons may enhance responses.^38^ ECP is generally well tolerated, but should be used with caution in patients with significant cardiac failure or recent ischemic events.

### Low‐dose MTX

Weekly low‐dose MTX (5–35 mg) is an effective, low‐toxicity option, particularly for erythrodermic MF, with higher ORR and PFS compared with stage IIB disease.^48^ Combination with IFN or bexarotene may improve response rates, though superiority over monotherapy is unproven.^49^ MTX is well tolerated and suited for older patients. It can be administered either orally or subcutaneously, and folic acid supplementation (e.g., 5 mg) is recommended on a non‐MTX day.

### Chemotherapy

Single‐agent chemotherapy (pegylated liposomal doxorubicin or gemcitabine) and polychemotherapy (CHOP or CHOP‐like) are generally discouraged as first‐line options, due to inferior survival outcomes in the CLIC study.^57^ However, pegylated doxorubicin or gemcitabine may be considered in patients with extensive nodal (N3) or tumoral disease (stage IIB) who are ineligible for BV, particularly as a bridge to transplantation.^54^

### Alemtuzumab

Alemtuzumab, targeting CD52, is active in erythrodermic and leukemic variants.^25^ Standard dosing (90 mg/week for up to 12 weeks) carries high infection and cytopenia risk; lower‐dose subcutaneous regimens achieve comparable efficacy, with improved tolerability.^26^, ^27^ Strict long‐term infection prophylaxis and monitoring are mandatory, given the risk of opportunistic infections and prolonged immunosuppression. Alemtuzumab is currently not commercially available for MF/SS in many countries due to changes in the manufacturer's development program. Access may therefore be limited to compassionate‐use or named‐patient programs, which may restrict its availability despite its potential role in selected high‐risk or refractory patients, particularly those with SS.

## MANAGEMENT OF RELAPSED OR REFRACTORY ADVANCED‐STAGE MF

Patients are considered refractory to a specific treatment if they show no or minimal response, or if the disease progresses despite therapy. Conversely, in cases of relapse following a successful first‐line treatment, the same therapy can often be reintroduced. Therefore, second‐line treatment options should generally be reserved for patients who are refractory to first‐line therapies, have contraindications to them, or experience insufficiently durable responses.

In recent years, the therapeutic landscape of advanced‐stage MF has evolved substantially with the introduction of targeted agents such as mogamulizumab and brentuximab vedotin (BV), both approved for patients previously treated with at least one systemic therapy and now considered key components of second‐ and subsequent‐line treatment strategies. In addition, other agents, including histone deacetylase inhibitors, have expanded the therapeutic armamentarium for relapsed or refractory disease, although their clinical use and availability remain more limited.

### BV

BV, an anti‐CD30 antibody–drug conjugate, is approved for adult patients with CD30 expression after at least 1 prior systemic therapy. It demonstrates substantial activity across advanced stages, especially in large‐cell transformation.^28^, ^29^, ^30^, ^31^ The standard schedule is 1.8 mg/kg intravenously every 3 weeks for up to 16 cycles. An alternative weekly schedule (1.2 mg/kg, administered weekly for 3 of every 4 weeks) has been explored in retrospective series and was associated with comparable response rates and potentially improved tolerability, with a lower incidence of clinically significant neuropathy.^32^ Weekly dosing may be considered in patients with more rapidly progressive or symptomatic disease, although prospective validation is lacking. Peripheral neuropathy is the most frequent adverse event and may require dose reduction (e.g., to 1.2 mg/kg), extension of dosing intervals (e.g., every 4–6 weeks), or treatment discontinuation. Neuropathy is often at least partially reversible upon treatment interruption or discontinuation, although complete resolution may not occur in all patients. Dose and AE profile are summarized in Table 7.

### Mogamulizumab

Mogamulizumab, a humanized anti‐CCR4 antibody, is approved for advanced MF/SS refractory to prior systemic therapy, particularly in patients with blood involvement. After weekly loading dose for the first cycle, it is administered every 2 weeks at a dose of 1 mg/kg. It significantly improves PFS and induces meaningful skin and blood responses.^50^ Real‐world data from the PROCLIPI cohort also suggest a potential overall survival benefit in appropriately selected patients.^51^ Responses are typically more pronounced in the skin and blood compartments, whereas nodal responses appear less consistent; therefore, radiologic monitoring is recommended to detect progression within the nodal compartment. The development of mogamulizumab‐associated rash (MAR) has been correlated with improved clinical outcomes.^52^, ^58^ Treatment is generally continued until disease progression, relapse, or unacceptable toxicity, although individualized treatment spacing or discontinuation after durable complete remission may be considered in selected cases.^59^ Its efficacy in stage IIB or in patients without blood involvement appears more limited.^53^ Caution is warranted in patients who are candidates for allogeneic HCT, as prior exposure to mogamulizumab has been associated with an increased risk of severe acute GVHD in other T‐cell malignancies. When allo‐HCT is planned, an appropriate wash‐out interval of at least 6 weeks between the last dose of mogamulizumab and transplantation should be considered.^60^, ^61^, ^62^ A recent study of 4 weekly mogamulizumab at an increased dose of 2 mg/kg has been shown to have a similar safety and plasma concentrations.^63^

### HDAC inhibitors

Vorinostat and romidepsin are FDA‐approved histone deacetylase inhibitors for relapsed or refractory MF after ≥1 prior systemic therapy.^42^, ^43^, ^44^ Romidepsin shows ORR 34%–45% in advanced‐stage MF, particularly tumor‐stage IIB, and SS. Romidepsin may be combined with other systemic agents to improve efficacy.^45^, ^46^ In contrast to the United States, no histone deacetylase inhibitors are currently approved by the European Medicines Agency (EMA) for MF/SS, and their use in Europe is therefore limited to off‐label settings or clinical trials. A recent maintenance trial of resminostat showed improved PFS compared to placebo. The median progression‐free survival (PFS) was 8.3 months (95% CI 4.2–15.7) in the resminostat group and 4.2 months (2.8–6.4) in the placebo group (HR 0.62 [95% CI 0.42–0.92]; P = 0.015).^47^

The following scenarios outline the management approach to systemic treatment in patients with relapsed or refractory advanced‐stage MF.

### Stage IIB—Relapse after first line

Stronger evidence is available for systemic retinoids, IFN‐α, TSEBI, BV, and mogamulizumab (Level II). In general, combination therapies tend to yield better response rates.^64^ Peg‐interferon‐α2a has demonstrated an ORR of 50% and a TTNT of 8.0 months, with higher doses of 180 μg weekly associated with better responses.^23^, ^65^ Therefore, combining it with bexarotene and PUVA may be considered.^23^, ^65^ Radiation therapy should be used as localized‐field treatment for a low number of lesions (e.g., up to 3 nodules), and as TSEBI for diffuse skin involvement.^13^ In stage IIB patients with only a few nodules, the disease can often be well controlled with low‐dose RT and pegylated interferon. Both of these options can be combined with systemic treatments, such as BV for CD30+ disease, to enhance the response.^66^ In patients with a limited number of tumors, intralesional BV has also shown high local efficacy, rapid responses, and an excellent safety profile, representing a practical and resource‐sparing option in selected cases.^67^ Notably, BV has been shown to improve response rates and PFS irrespective of large‐cell transformation (LCT) status and should be considered a preferred option in patients with LCT. The role of mogamulizumab in the absence of blood involvement and in T3 disease is limited.^50^, ^52^, ^53^, ^68^, ^69^ Other options, supported by weaker evidence (Level IV), include low‐dose MTX (ORR 43.5%)^48^ and single‐agent chemotherapy regimens (ORR 41%–84% for pegylated liposomal doxorubicin and 48%–70% for gemcitabine).^40^, ^54^, ^70^, ^71^, ^72^ Overall, pegylated liposomal doxorubicin and gemcitabine appear to be the most commonly used single‐agent chemotherapies in this setting. Pegylated liposomal doxorubicin may also be considered as a maintenance strategy in selected patients achieving disease control, particularly when prolonged treatment is clinically indicated and well tolerated.^73^

### Stage IIB—Refractory to first line

In patients with stage IIB disease refractory to first‐line therapy, BV should be considered in the presence of CD30 expression, even at low or minimal levels, as clinical responses have been observed irrespective of a defined expression cut‐off. Real‐world data also suggest that retreatment with BV may be feasible and clinically beneficial in selected patients who previously responded and subsequently relapsed. The selection of subsequent chemotherapy regimens, whether CHOP‐based or single‐agent therapy, should be discussed in a multidisciplinary tumor board, involving dermatologists, hematologists, and radiation oncologists, taking into account the CLIPI risk scores,^1^ the patient's comorbidities, age, and the potential for subsequent allogeneic hematopoietic cell transplantation (allo‐HCT) (see Section 6 for detailed recommendations). In general, single‐agent chemotherapy is preferred because of its more favorable toxicity profile, particularly in older or comorbid patients. Available options include gemcitabine,^40^, ^41^, ^57^ pegylated liposomal doxorubicin,^54^, ^57^ and, in selected cases, pralatrexate, although the latter is not approved in Europe for MF/SS.^55^, ^56^ Combination regimens may be considered in selected cases requiring rapid disease control, especially when bridging to allo‐HCT is planned.^74^, ^75^ The choice between combination chemotherapy and single agents should therefore also consider donor availability and the patient's willingness to proceed to transplantation after achieving disease control. Therefore, discussion of allo‐HCT should be encouraged in patients with advanced‐stage CTCL and LCT, irrespective of the line of therapy^61^, ^62^, ^76^ (see Section 6 for detailed allo‐HCT guidance). To date, no head‐to‐head comparison of single‐agent versus polychemotherapy has been conducted, and there is no strong evidence supporting the superiority of combination regimens over single agents as a bridge to transplantation. Pretransplant TSEBI (e.g., 24 Gy) should be considered as part of the allo‐HCT strategy, with the aim of achieving deeper disease control prior to transplantation (see Section 6 for transplant preparation and monitoring details).^13^, ^61^, ^62^

### Stage III—Relapse after first line

Stronger evidence is available for systemic retinoids, IFN‐α, mogamulizumab, and TSEBI (Level II). Combination therapies, particularly those incorporating ECP and phototherapy‐based regimens with agents like retinoids and IFN‐α, are recommended. Notably, no significant differences in response rates have been demonstrated between different retinoid molecules.^20^, ^22^, ^77^ Low‐dose MTX may be considered for stage IIIA patients due to its favorable safety profile and symptom‐control efficacy, with reported ORR of up to 61%, despite no effect on the circulating neoplastic clone.^48^ After failure of a prior systemic therapy, mogamulizumab should be considered as a solid option, with ORR rates of 23% in stage III patients.^78^ Data on BV are less robust, as only four patients were included in the ALCANZA trial, though two achieved an ORR4. Regardless of concomitant therapy, TSEBI offers a valid option to complement systemic treatment, providing good symptom control, with an ORR of 50%.^79^

### Stage III—Refractory to first line

In patients with refractory disease, mogamulizumab should be considered in those not previously treated with this agent. If CD30 expression is present, BV may be another viable option. The selection of subsequent chemotherapy regimens should be discussed in a multidisciplinary tumor board, including dermatologists, hematologists, and radiation oncologists, taking into account CLIPI risk scores, and the patient's comorbidities, age, and potential for subsequent allo‐HCT (refer to Section 6 for comprehensive recommendations).^1^, ^61^, ^62^, ^76^ In stage III, there are insufficient data to support the use of multimodal chemotherapy regimens. Therefore, single‐agent chemotherapy options, such as gemcitabine^40^, ^41^, ^57^ or pegylated liposomal doxorubicin,^54^, ^57^ may be considered.^54^, ^73^ Although not approved in Europe for MF/SS, pralatrexate, a folate analogue metabolic inhibitor structurally related to MTX, may represent a reasonable therapeutic option in selected previously treated patients with relapsed/refractory disease.^55^, ^56^

## TREATMENT ALGORITHMS FOR SS (FIRST LINE AND SUBSEQUENT LINES)

### First‐line therapy

First‐line treatment of SS is mainly based on immunomodulatory and biologic agents that target both skin and circulating malignant T‐cells. The primary goals are to induce remission, control symptoms, limit infectious complications, and preserve quality of life, while maintaining options for subsequent therapy or transplantation.

The choice of initial therapy depends on the extent of skin and blood involvement, comorbidities, and local drug availability. In most cases, combination regimens integrating ECP, retinoids, and/or IFN‐α represent the standard of care.

A proposed therapeutic algorithm for the management of patients with SS is shown in Figure 1.

**Figure 1:**
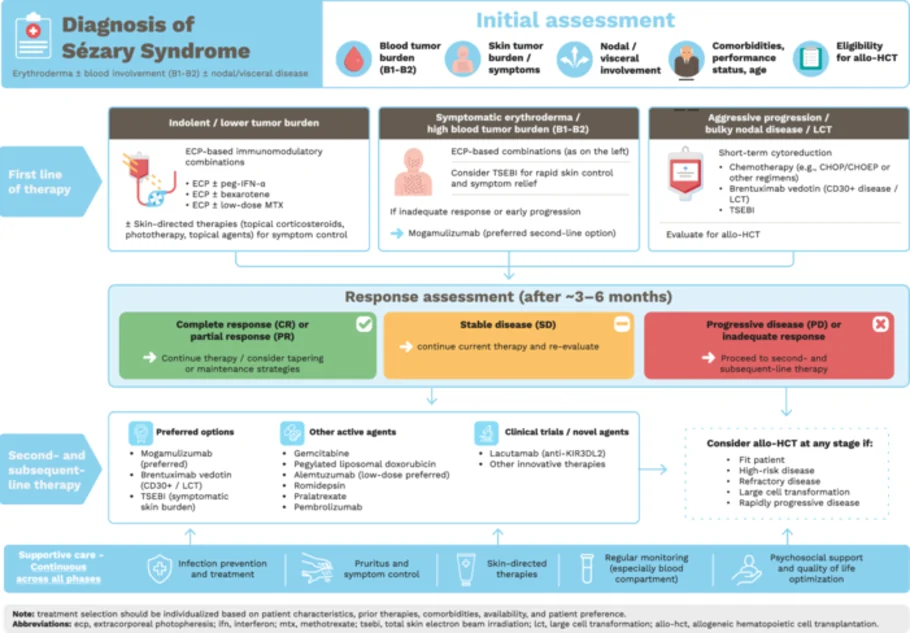
Proposed therapeutic algorithm for patients with Sézary syndrome.

**ECP** is considered the cornerstone of first‐line therapy and is particularly effective in patients with both erythroderma and circulating Sézary cells. Responses are often gradual but durable, with ORRs ranging from 54% to 77%, and excellent tolerability.^33^, ^80^ The blood response usually occurs earlier than the skin response and is maintained significantly longer.^81^ ECP may be administered as monotherapy or combined with systemic agents—most commonly bexarotene or IFN‐α—to improve efficacy and achieve deeper blood responses. While ECP is associated with low systemic toxicity, its use requires particular attention in patients with clinically significant cardiac disease, especially those with recent ischemic events.

**Retinoids and rexinoids**, particularly bexarotene, act by inducing apoptosis and modulating T‐cell differentiation. In SS, they are frequently combined with ECP or IFN‐α to enhance skin responses and improve disease control. Bexarotene is generally well tolerated, although it requires pre‐emptive management of metabolic adverse effects, including routine thyroid hormone replacement and lipid‐lowering therapy, together with close monitoring of lipid and thyroid function.^19^

**IFN‐α** (currently pegylated IFN‐α2a or ropeginterferon‐α2b) remains a valuable and widely used therapy in SS, capable of inducing partial or complete responses in a substantial proportion of patients. It may be administered alone or in combination with ECP or bexarotene, and is particularly helpful for controlling erythroderma and pruritus.

**Chemotherapy** Single‐agent chemotherapy is generally preferred over multi‐agent regimens because of its more favorable toxicity profile. Combination chemotherapy (e.g., CHOP or CHOEP) may be considered in cases of aggressive progression or refractory disease, but should generally be reserved for short‐term tumor debulking due to toxicity and limited durability of response.

**Topical corticosteroids** and other SDTs serve mainly as adjuncts for symptom control, improving erythema and pruritus, but rarely providing sufficient systemic benefit as monotherapy.

### Second‐ and subsequent‐line therapies

Therapy selection after first‐line failure is individualized according to patient comorbidities, prior responses, and transplant eligibility.

**Mogamulizumab** represents the preferred second‐line option for SS. In the phase III *MAVORIC* trial,^50^ mogamulizumab significantly improved PFS (7.7 vs. 3.1 months) and ORR (37% vs. 2.3%) compared with vorinostat, with the greatest benefit observed in patients with B2 blood stage involvement. Real‐world data from the OMEGA registry confirmed high efficacy (ORR 69.5%) and durable responses,^52^ and treatment with mogamulizumab has been significantly associated with reduced mortality in patients with SS (HR 0.30, P = 0.013). Blood involvement at the B2 level often responds rapidly, with normalization of circulating Sézary cells frequently observed after the first treatment cycle.^50^ MAR occurs in approximately 25% of patients and correlates with a favorable outcome.^52^, ^58^ Treatment is usually continued until progression or intolerance; interval extension or discontinuation after complete remission may be considered.^59^, ^69^, ^82^ Regular flow‐cytometric monitoring every 3 months is recommended.

**BV**, targeting CD30, showed a superior ORR4 (50% vs. 10%) and median PFS (16.7 vs. 3.5 months) over the physician's choice in the *ALCANZA* trial.^31^ Although SS was not included, real‐world series report response rates >70%.^83^ BV may be particularly useful in CD30^+^ or large‐cell–transformed disease but requires careful monitoring for peripheral neuropathy. Treatment is generally administered for a maximum of 16 cycles, in line with the ALCANZA protocol.

**Chemotherapy** offers transient benefit and is reserved for patients refractory to targeted or biologic agents. Single‐agent gemcitabine or pegylated liposomal doxorubicin provides modest responses, with median time‐to‐next treatment <5 months.^64^

**TSEBI** may rapidly reduce cutaneous tumor burden and improve symptoms.^13^ Low‐dose regimens are well tolerated and can serve as bridging therapy before other systemic treatments.

**Alemtuzumab**, a CD52‐targeting monoclonal antibody, achieves ORR of 30%–40% in heavily pretreated SS but carries the risk of severe infections and cytopenias.^25^, ^84^ Low‐dose regimens improve safety and may yield higher CR rates.^85^ Its use is best reserved for refractory patients under strict infectious monitoring.^86^

**Other options** include histone deacetylase inhibitor romidepsin, antifolate pralatrexate, and immune checkpoint inhibitors (pembrolizumab). Lacutamab, an anti‐KIR3DL2 antibody, demonstrated high efficacy and tolerability in mogamulizumab‐refractory SS;^87^ however, at present, its use is restricted to clinical trial settings.

In patients with SS, consideration of allo‐HCT should be based on overall disease risk and patient eligibility^61^, ^62^, ^76^ rather than strictly on treatment line, and is discussed in detail in Section 6.

### Maintenance therapy

Maintenance aims to prolong remission using well‐tolerated systemic or SDTs. While no standard regimen exists, ECP, MTX, IFN‐α, and bexarotene have been used effectively for years.^88^ Mogamulizumab maintenance may be considered in patients with persistent blood involvement and in patients under remission off therapy. In patients achieving remission off therapy, rechallenge at relapse is a feasible option.^82^ The RESMAIN trial demonstrated the value of maintenance: resminostat, an oral HDAC inhibitor, significantly improved PFS (8.3 vs. 4.2 months) versus placebo.^47^ However, resminostat was denied market approval by the EMA in May 2025, on the basis that the benefits did not outweigh the risks.^89^ Future strategies will likely incorporate biomarker‐guided or molecularly targeted maintenance approaches.

## COMBINATION THERAPY

### Introduction

In advanced‐stage MF, combination treatment (CoT) is generally considered a second‐line approach after failure of first‐line therapy. The choice of treatment is complex and depends on the type of skin involvement (ulcerated plaques, nodules, tumors, or erythroderma) as well as the presence of nodal or blood involvement^12^, ^90^ (Table 8). The overarching goal of CoT is to improve both the depth and the duration of response across all compartments: skin, blood, and lymph nodes.

**Table 8:** Combination therapies by stage.

Stage of disease	Common combination therapies	Notes
Advanced‐Stage MF (IIB)	PUVA + Oral Bexarotene	TSEBI is an alternative option where available and accessible.
	PUVA + Peg‐IFN‐α	TSEBI is an alternative option
	PUVA + Peg‐IFN‐α +/‐ Limited Field, low‐dose Radiotherapy IFN‐α2b + low‐dose MTX IFN‐α2b + retinoids	TSEBI is an alternative option.
	TSEB 12 Gy + Mogamulizumab	Under investigation in ongoing trials (EORTC Mogat).
Advanced‐Stage MF (III: IIIA, IIIB)	ECP + Low‐dose MTX	TSEBI is an alternative option.
	ECP + Oral Bexarotene	TSEBI is an alternative option.
	ECP + Peg‐IFN	TSEBI is an alternative option.
	ECP + PUVA IFN‐α2b + low‐dose MTX IFN‐α2b + retinoids	TSEBI is an alternative option.
	Mogamulizumab + ECP	Used in clinical practice.
	Mogamulizumab + TSEB	Under investigation in ongoing trials (Stanford).
Sézary Syndrome (SS) (IVA)	ECP + Low‐dose MTX	Strong indication for low tumor burden patients.
	ECP + Oral Bexarotene	Strong indication for low tumor burden patients.
	ECP + Peg‐IFN	Strong indication for low tumor burden patients.
	Mogamulizumab + ECP	Used for both high tumor burden patients and low tumor burden patients refractory to other combinations. Can be used with induction and maintenance or simultaneously.
	TSEB 12 Gy + Mogamulizumab	Under investigation in ongoing trials (US Stanford).

A key consideration concerns the efficacy of available CoT regimens relative to monotherapy. Notably, some commonly used combination approaches lack robust supporting evidence. In this regard, it is recommended to distinguish between induction and maintenance phases: patients achieving clinical benefit during induction may be considered for maintenance therapy, often involving tapering of treatment regimens to optimize the duration of response.

Other important questions include the optimal timing and sequencing of different CoT regimens, which should be tailored to the individual patient, and whether dosage adjustments are necessary when treatments are combined. Currently, data on these aspects are limited, highlighting the need for careful clinical judgment and individualized decision‐making.

### Combination therapies in advanced‐stage MF

In stage IIB MF, combination therapy (CoT) with PUVA plus oral bexarotene and/or peg‐IFN‐α, with or without limited‐field RT targeting individual lesions, may represent a valid therapeutic option alongside TSEBI, particularly in patients in whom a systemic immunomodulatory approach is preferred or when individualized treatment strategies are required.^13^

Low‐dose MTX in combination with retinoids is another viable option; dose adjustments of both agents are recommended to minimize toxicity.^12^ Other reported effective combinations include IFN‐α 2b with low‐dose MTX or retinoids.^91^

CoT may also be considered as an alternative or bridge to monotherapy with BV (suitable option, depending on CD30 expression) or mogamulizumab.^12^, ^90^ Additional combinations under investigation include mogamulizumab with ECP^92^ and mogamulizumab with low‐dose (12 Gy) TSEBI (ongoing trials, Stanford and EORTC CLTG [Mogat]).

Data on the efficacy and safety of CoTs involving BV or mogamulizumab remain limited, and further studies are needed to establish optimal regimens and sequencing strategies.

### Combination therapies in SS

In stage IVA SS, combination therapy (CoT) with ECP plus pegylated interferon‐α (peg‐IFN‐α), low‐dose MTX, with or without oral low‐dose bexarotene, is commonly indicated in patients with low tumor burden. Mogamulizumab is recommended for patients with high tumor burden, as well as for those with low tumor burden who are refractory to ECP combined with low‐dose MTX and/or oral bexarotene. BV shows limited efficacy in SS and may be considered as a second‐line option, independent of CD30 expression.^12^, ^90^ Any combinations containing ECP were found to be more effective in prolonging TTNT than non‐ECP‐containing regimes.^64^

Additionally, antibiotic therapy may play a supportive role in both advanced MF and SS, as it can contribute to disease control in selected patients with treatment‐refractory disease^93^ and because *Staphylococcus aureus* colonization has been shown to promote drug resistance in malignant T‐cells.^94^

Infection represents a major cause of morbidity and mortality in advanced MF/SS. Therefore, prompt identification and treatment of *S. aureus* colonization, and overt infection are essential components of supportive care and may contribute toward reducing infectious complications and overall morbidity.^93^, ^95^

Given the increased risk of infectious complications in heavily pretreated and immunocompromised patients with advanced MF/SS, antibacterial, antiviral, and/or anti‐Pneumocystis, prophylaxis should be considered in selected patients receiving highly immunosuppressive therapies, particularly alemtuzumab‐based regimens or combination cytotoxic treatments.

## ALLOGENEIC HEMATOPOIETIC CELL TRANSPLANTATION (ALLO‐HCT)

Allo‐HCT remains the only potentially curative option for patients with advanced CTCL, including MF and SS, primarily through the graft‐versus‐lymphoma (GvL) effect. However, it is associated with significant morbidity and mortality, with nonrelapse mortality (NRM), reported at approximately 15%–20%.^76^, ^96^ Notably, more recent series suggest lower NRM rates, around 10%, likely reflecting improvements in patient selection, supportive care, and transplant strategies, including reduced‐intensity conditioning and optimized graft‐versus‐host disease prophylaxis.^97^, ^98^, ^99^ In a propensity score‐matched prospective study, allo‐HCT was shown to improve both PFS and OS.^97^, ^98^ Although relapse occurs in up to 50% of patients—often at a low grade or indolent level—durable complete remissions can be achieved in a substantial proportion of individuals who have undergone transplantation.^99^, ^100^ Updated consensus recommendations from the EBMT Practice Harmonisation & Guidelines Committee, jointly published in *Bone Marrow Transplantation* ^61^ and *JEADV,* ^62^ now provide unified guidance on patient selection, timing, and transplant strategy.

### Indications and eligibility

Allo‐HCT should be considered for patients with advanced or high‐risk CTCL (MF stage IIB–IV and SS) who have relapsed or are refractory after at least one systemic therapy, or who present poor‐prognostic features such as large‐cell transformation, N3 nodal or M1 visceral disease, or elevated serum LDH.^1^, ^8^, ^101^, ^102^, ^103^, ^104^, ^105^ For these patients, early referral to a transplant center is essential, ideally at the time a first systemic therapy is being planned, in order to assess transplant eligibility and initiate donor search procedures.

Patients with early‐stage IA–IIA MF are generally not candidates, though highly selected relapsed and refractory IB–IIA cases may be evaluated individually.^60^, ^61^, ^62^ No fixed number of prior therapies is mandated; the decision should balance disease trajectory, age, comorbidities, and expected quality of life.

### Disease status before transplant

Best outcomes are achieved when transplantation is performed during complete or very good partial remission (CR/VGPR), ideally with clearance of skin and nodal disease. Patients with limited residual blood involvement can still achieve durable remission via GvL effects. Conversely, transplantation in refractory or rapidly progressive disease is discouraged. Pre‐transplant bridging should aim to minimize tumor burden using available modalities such as mogamulizumab, BV, TSEBI, or multi‐agent chemotherapy in transformed MF.^60^, ^61^, ^62^, ^76^, ^99^, ^106^, ^107^, ^108^

### Sequencing and wash‐out

Both mogamulizumab and PD‐1 inhibitors may increase the risk of acute or steroid‐refractory graft‐versus‐host disease (GVHD).^109^, ^110^, ^111^ A minimum wash‐out of 2–4 months after mogamulizumab and ≥6 weeks after checkpoint inhibitors is recommended when clinically feasible.^112^ Nevertheless, the use of mogamulizumab to achieve optimal blood remission before allo‐HCT remains appropriate when balanced with timing considerations.

### Conditioning and donor selection

Reduced‐intensity conditioning (RIC) regimens are preferred, offering similar efficacy with lower NRM than myeloablative conditioning.^60^, ^61^, ^62^, ^76^ Although the EBMT transplant conditioning intensity (TCI) score has not been validated specifically in MF/SS, it may provide a useful framework for regimen selection. The TCI score stratifies conditioning regimens based on components and dose intensity, classifying them as low, intermediate, or high intensity.^113^ The curative benefit derives primarily from the GvL effect rather than cytotoxic intensity. Conditioning can be complemented by low‐dose TSEBI to clear residual skin lesions.

The use of alemtuzumab‐containing conditioning regimens or GVHD prophylaxis platforms deserves particular consideration in MF/SS. While in vivo T‐cell depletion may reduce the incidence and severity of GVHD, it may also attenuate the GvL effect and increase infectious risk. Therefore, the choice of T‐cell depletion strategy should be individualized, balancing disease control, GVHD risk, and immune reconstitution.

Donor hierarchy follows general lymphoma standards: matched related donor (MRD) > matched unrelated (MUD) > haploidentical > mismatched MUD. Peripheral blood stem cells are most used, though bone marrow grafts may be preferred in haploidentical settings.^60^, ^61^, ^62^

### Posttransplant considerations

Relapse remains the leading cause of treatment failure, often limited to the skin. Many patients achieve secondary remissions through immunosuppression tapering, donor lymphocyte infusion (DLI), or interferon‐based therapy, emphasizing the persistence of a GvL effect.^60^, ^61^, ^62^, ^76^, ^99^ Low‐grade relapses may be treated with SDTs.^75^ In clinical practice, posttransplant relapses are often treated similarly to those occurring in nontransplanted patients, including chlormethine gel, PUVA, bexarotene, ECP, or low‐dose MTX, while carefully balancing immunosuppression and the risk of GVHD. Local RT or TSEB irradiation represent other effective options to achieve new remission in patients relapsing after allo‐HCT.^99^ Routine measurable residual disease (MRD) monitoring by TCR sequencing is not yet validated, but may be informative within clinical trials.^114^

## NOVEL SYSTEMIC THERAPEUTIC APPROACHES FOR TREATING ADVANCED CTCL

Improved understanding of the pathogenesis of cutaneous lymphomas has led to consideration of many potential therapeutic options discussed below. The agents discussed in this section represent investigational or early‐phase approaches with potential activity in CTCL; however, available data are still limited and not yet mature. Their role in routine clinical practice remains to be defined.

### Lacutamab

Lacutamab is a humanized anti‐KIR3DL2 antibody that targets KIR3DL2 expressed in 85% of SS patients. The TELLOMAK study evaluated Lacutamab in RR MF or SS patients after 2 previous lines of treatment, including mogamulizumab for SS.^115^ The ORR Global response rate for SS was 42.9%, with a median duration of response of 25.6 months, with manageable side effects.^115^

### CTX‐130

CTX‐130 is a CD70‐directed, allogenic CRISPR‐Cas9‐engineered CAR T‐cell therapy currently being evaluated in patients with T‐cell malignancies, including CTCL. In the phase 1 COBALT‐LYM study, 39 heavily pretreated patients with relapsed or refractory peripheral T‐cell lymphoma (PTCL) or CTCL were treated.^116^ Of these, only 10 had CTCL. Overall, CTX130 demonstrated manageable safety and preliminary efficacy, with an objective response rate (ORR) of 46.2% across the entire cohort, increasing to 51.6% at higher dose levels (dose ≥3), including 19.4% complete responses. In the CTCL subgroup, the ORR was 40%, with complete responses observed in 10% (1/10) of patients.^116^ Cytokine release syndrome occurred in 67% of patients (mostly grade 1–2), and grade 3–4 cytopenias were common. Infections represented the most frequent adverse events. Serious adverse events occurred in 64% of patients, although dose‐limiting toxicities were rare.^116^ The long‐term efficacy of CTX‐130 in CTCL remains to be determined.

### Fenretinide

Fenretinide binds to retinoic acid receptors (RARs) and retinoid X receptors (RXRs), and modulates gene expression.^117^ ST‐001 is a nanoFenretinide, composed of phospholipid nanoparticles, that improves tolerability to allow therapeutic doses to be administered. An ongoing phase 1a/1b study is evaluating this agent in relapsed/ref T‐cell lymphomas including CTCL.^118^

### JAK/STAT inhibitors

A phase II clinical study evaluating ruxolitinib in PTCL and CTCL cohorts reported ORRs of 38% and 40% in patients enriched for JAK/STAT mutations or with evidence of pathway activation.^119^ However, activity in CTCL was limited: only 1 of 7 patients achieved a partial response, with no complete responses observed. In the original report, ruxolitinib was considered unlikely to be effective as a single agent in this setting.^120^

Other agents targeting the JAK/STAT pathway are currently under investigation;^121^ nevertheless, available data remain preliminary, and responses in CTCL have generally been modest and not consistently durable. Further studies are needed to better define the role of this class in advanced disease, especially due to the emerging reports on the cutaneous worsening including stage progression in patients treated with JAK inhibitors for presumed generalized dermatitis‐onset atopic dermatitis who eventually were diagnosed with MF, the majority with advanced‐stage MF or SS.^122^

### EZH2 inhibitors

EZH2 is an enzyme that is overexpressed in T‐cell lymphomas.^123^ EZH2 inhibitors have shown efficacy in T‐cell lymphomas, with a very favorable safety profile.^124^ Several oral agents in this class are being evaluated.^125^

### PI3kinase inhibitors

PI3K inhibitors, particularly those targeting the delta isoform, have shown activity in CTCL, including MF and SS. In early‐phase studies including patients with CTCL, duvelisib demonstrated an ORR of approximately 31%. Although higher response rates have been reported in PTCL cohorts (e.g., ORR 48% in the PRIMO study),^126^ these results cannot be directly extrapolated to MF/SS populations. Other similar agents including Tenalisib^127^ and Linperlisib^128^ have also been evaluated with preliminary signals of activity either as monotherapy or in combination regimens.^129^, ^130^ However, available data remain limited, and the durability of responses requires further investigation.

### CCR4 antagonists

CCR4 is highly expressed in CTCL cells and represents a potential therapeutic target. Small‐molecule CCR4 antagonists, including C021 and AZD2098, have demonstrated preclinical activity in vitro and in vivo, and may warrant further evaluation in clinical trials.^131^

### CCR8 monoclonal antibodies

CCR8 is expressed at the surface of intratumor regulatory T‐cells and in the skin of CTCL patients,^132^ and several anti‐CCR8 antibodies are in development in the treatment of solid cancers. One of them has demonstrated an acceptable safety profile and preliminary antitumor activity in CTCL patients.^133^ Another one, DT‐7012, is entering phase 1 in CTCL patients (NCT07213882).^134^

### CD47/SIRPα

The interaction between CD47 and SIRPα plays a significant role in cancer immune escape and inflammation. A trial using intralesional TTI‐621 in RR CTCL and SS has been completed with no safety concerns and a decrease in the Composite Assessment of Index Lesion Severity (CAILS) score of at least 50% in 34% of patients.^135^ In addition, in a phase I study of systemic TTI‐621 including 29 patients with CTCL (24 MF and 5 SS), objective responses were observed in approximately 20% of patients, further supporting the clinical activity of CD47‐targeting strategies in this setting.^136^ Agents targeting this pathway are currently under clinical investigation.

### CDK9 inhibitors

Cyclin‐dependent kinase 9 (CDK9), in partnership with cyclin T, regulates cell cycle progression and has been shown to be a driver of CTCL. Initial results with CDK9 inhibitors were disappointing, but combination strategies may be promising.^137^

### CD74 ADC

CD74 is widely expressed in MF and SS. STRO‐001 is a novel anti‐CD74 ADC, containing 2 non‐cleavable maytansinoid linker warheads per molecule. It has been shown to efficiently kill CTCL‐derived cell lines and eradicates murine xenotransplants of CTCL cell lines in vivo.^138^

### Denileukin diftitox

Denileukin diftitox‐cxdl (DD‐cxdl), a reformulated and higher‐purity version of denileukin diftitox targeting the IL−2 receptor, has recently been evaluated in a phase III, multicenter, single‐arm trial in heavily pretreated patients with relapsed/refractory stage IA–IIIB MF/SS.^139^ In the primary efficacy analysis set (*n* = 69), the ORR was 36.2%, including 8.7% complete responses, with a median duration of response of 8.9 months and a median time to response of 1.4 months. Most patients (84%) experienced a reduction in skin tumor burden. Treatment‐related adverse events were frequent but generally manageable; capillary leak syndrome occurred in 20% (grade ≥3: 5.8%). These data supported US FDA approval in 2024 for patients with R/R stage I–III CTCL after at least one prior systemic therapy.^139^

### PTX‐100

PTX‐100 is a novel inhibitor of geranylgeranyl transferase‐I (GGTase‐I), a key regulator of Rho/Ras signaling pathways implicated in malignant T‐cell proliferation and survival. Early‐phase clinical studies in relapsed/refractory T‐cell lymphomas, including CTCL, have shown preliminary evidence of antitumor activity with manageable toxicity. The US FDA has granted PTX‐100 Fast Track Designation for the treatment of adults with relapsed or refractory MF. Further clinical evaluation is ongoing to better define the efficacy and safety of this agent in advanced MF/SS.^140^

### Conclusions

The above description is not exhaustive. Multiple additional strategies are currently under investigation to develop novel treatments for CTCL and improve patient outcomes.

## FOLLOW‐UP, LONG‐TERM IMPLICATIONS, AND SURVIVORSHIP

In the clinical management of advanced MF patients, as well as other CTCL patients, a multidisciplinary approach is mandatory for the development of integrated care, with the integration of the different medical expertise such as dermatologists, pathologists, radiation oncologists, hematologists, and other health care professionals involved in this disease field.^90^

The management of patients with advanced‐stage MF and SS extends, however, beyond therapeutic interventions. Long‐term follow‐up strategies must account for the risk of relapse, treatment‐related toxicities, second malignancies, psychosocial consequences, and overall survivorship. With improved treatment outcomes and longer survival, structured follow‐up protocols and survivorship care plans are essential for optimizing QoL and functional outcomes.

Advanced‐stage MF and SS significantly impair QoL due to chronic skin symptoms, immune dysfunction, psychological burden, and cumulative treatment‐related effects. Pruritus, fatigue, infections, disfigurement, and psychosocial distress are key contributors to reduced functional well‐being.^141^

While factors such as age and disease duration do not appear to influence QoL, the literature presents conflicting results regarding the impact of female gender.^141^ In clinical practice, QoL questionnaires (e.g., DLQI or Skin‐index 29) should be used to assess QoL impairment, although their comparability with benign dermatological conditions is limited. Since pruritus is the most bothersome symptom and its relief is linked to improved QoL, every effort should be made to maximize its control.^142^ A visual analog scale or other validated tool is highly recommended to assess the severity of pruritus in daily clinical practice.

Since pruritus is the most burdensome symptom and its relief is consistently associated with improved QoL, systematic assessment and proactive management are essential. A visual analog scale or other validated instruments should be routinely used to quantify itch severity and monitor response to treatment. Management requires a multimodal approach, including optimization of disease control, SDTs, antihistamines (although often insufficient), gabapentinoids, mirtazapine, or other neuromodulatory agents when appropriate. Emerging therapies targeting IL‐31 signaling, such as nemolizumab, represent promising investigational options for severe, refractory pruritus.^143^

In addition, patients with advanced MF/SS frequently develop ulcerated tumors, erosions, and chronic wounds, which increase the risk of infection, pain, and social isolation. Structured wound care protocols—including regular cleansing, non‐adherent dressings, infection surveillance, and appropriate use of topical antimicrobials—are critical components of supportive management. Close collaboration with dermatology nurses and wound‐care specialists may substantially improve symptom control, reduce complications, and enhance overall quality of life.

### Conclusions

Follow‐up and survivorship care in CTCL require a comprehensive, multidisciplinary, and patient‐centered approach. The CLIPI risk score may help predict survival and identify high‐risk patients who could benefit from closer monitoring and timely therapeutic escalation. Decisions regarding allogeneic bone marrow transplantation should be considered early in the disease course to allow optimal planning for long‐term disease control. Imaging should be used judiciously according to the clinical stage and symptoms. Improving QoL and minimizing treatment‐related sequelae are as important as controlling disease burden, particularly as novel therapies continue to extend survival in this population.

## AUTHOR CONTRIBUTIONS

**Francesco Onida**: Conceptualization; methodology; supervision; project administration; writing—original draft; writing—review and editing. **Pietro Quaglino**: Conceptualization; methodology; writing—original draft; writing—review and editing; supervision. **Julia Scarisbrick**: Writing—original draft; writing—review and editing; conceptualization; methodology. **Anna Sureda**: Conceptualization; methodology; writing—original draft; writing—review and editing. **Emmilia Hodak**: Conceptualization; methodology; writing—review and editing; writing—original draft. **Evangelia Papadavid**: Conceptualization; methodology; writing—original draft; writing—review and editing. **Silvia Alberti‐Violetti**: Writing—original draft; writing—review and editing. **Martine Bagot**: Writing—original draft; writing—review and editing. **Lorenzo Cerroni**: Writing—original draft; writing—review and editing. **Amrita Goyal‐O'Leary**: Writing—original draft; writing—review and editing. **Rudolf Stadler**: Writing—original draft; writing—review and editing. **Rein Willemze**: Writing—original draft; writing—review and editing. **Jasmine Zain**: Writing—original draft; writing—review and editing. **Marco Ardigò**: Writing—original draft; writing—review and editing. **Emilio Berti**: Writing—original draft; writing—review and editing; supervision. **Adèle de Masson**: Writing—original draft; writing—review and editing. **Rafael F. Duarte**: Writing—original draft; writing—review and editing. **Francine Foss**: Writing—original draft; writing—review and editing. **Eva González‐Barca**: Writing—original draft; writing—review and editing. **Joan Guitart**: Writing—original draft; writing—review and editing. **Pablo Ortiz‐Romero**: Writing—original draft; writing—review and editing. **Alessandro Pileri**: Writing—original draft; writing—review and editing. **Pierluigi Porcu**: Writing—original draft; writing—review and editing. **Henry M. Prince**: Writing—original draft; writing—review and editing. **Sima Rozati**: Writing—original draft; writing—review and editing. **Serena Rupoli**: Writing—original draft; writing—review and editing. **Maarten H. Vermeer**: Writing—original draft; writing—review and editing. **Sean Whittaker**: Writing—original draft; writing—review and editing. **Nicola Pimpinelli**: Conceptualization; methodology; writing—original draft; writing—review and editing; supervision. **Pier Luigi Zinzani**: Conceptualization; methodology; writing—original draft; writing—review and editing; supervision; project administration.

## CONFLICT OF INTEREST STATEMENT

Francesco Onida: − Honoraria*: Kyowa Kirin, Takeda, Menarini StemLine, Johnson&Johnson. − *Consultancy/Advisory Board*: Takeda. Pietro Quaglino: − Honoraria*: Kyowa Kirin, Takeda, Therakos, Helsinn‐Recordati, 4SC. − *Consultancy/Advisory Board*: Kyowa Kirin, Takeda, Therakos, Helsinn‐Recordati, 4SC, Celgene, Ideogen. − *Research funding/Grants*: Kyowa Kirin. − *Travel support:* Kyowa Kirin, Takeda, Helsinn‐Recordati. Julia Scarisbrick: − Honoraria*: Therakos, Recordati, Kyowa Kirin. − *Consultancy/Advisory Board*: Kyowa Kirin, Solgenix, Innate Pharma, HAC Pharma, 4SC. Anna Sureda: − Honoraria*: Takeda, BMS/Celgene, MSD, Janssen, Amgen, Novartis, Gilead Kite, Sanofi, Roche, GenMab, AbbVie, Jazz Pharmaceuticals, Sobi. − *Consultancy/Advisory Board*: Takeda, BMS/Celgene, Novartis, Janssen, Gilead Kite, Sanofi, GenMab, AbbVie, Miltenyi. − *Research funding/Grants*: Takeda. Emmilia Hodak: − Honoraria*: Rafa. − *Consultancy/Advisory Board*: Pfizer, Sanofi, Helsinn, Recordati, Medison, Teva. − *Travel support*: Rafa. Evangelia Papadavid: − *Consultancy*: Janssen, AbbVie, Novartis, UCB, Recordati. − *Speaker & Advisory Board*: Helsinn, Recordati, UCB, HC pharma. − *Research grants*: Recordati/Helsinn. − *Travel support*: Recordati, Novartis, AbbVie, Janssen. Silvia Alberti‐Violetti: − Honoraria*: Takeda, Kyowa Kirin, Recordati Rare Diseases, Incyte. − *Research funding/Grants*: Incyte, Recordati Rare Diseases, Pfizer, Kyowa Kirin. Martine Bagot: *Consultancy/Advisory Board/Travel support*: Kyowa Kirin, Helsinn‐Recordati, Galderma, Therakos. Lorenzo Cerroni: Nothing to disclose. Amrita Goyal‐O'Leary: Nothing to disclose. Rudolf Stadler: − Honoraria*: Kyowa Kirin, Takeda, Recordati Rare Diseases, 4SC, HAC Pharma. − *Consultancy/Advisory Board:* Kyowa Kirin, Takeda, Recordati Rare Diseases, 4SC, HAC Pharma. Rein Willemze: − Nothing to disclose. Jasmine Zain: − *Consultancy:* Secura Bio, Kyowa Kirin, Ascentage, Incyte, Dren Bio. − *Research support*: Kyowa Kirin, Incyte, Ascentage, Corvus. Marco Ardigò: − *Consultancy*: Pierre Fabre, Recordati Rare Diseases, Almirall, SunPharma, Regeneron. − *Speaker & Advisory Board*: Kyowa Kirin, Almirall, Incyte, Pierre Fabre, L'Oreal, Recordati Rare Diseases, Regeneron, SunPharma, Helsinn. Emilio Berti: Nothing to disclose. Adèle de Masson: − Honoraria*: Takeda, Kyowa Kirin, Helsinn, Recordati Rare Diseases, Almirall, Novartis. − *Research support*: Kyowa Kirin. − *Travel support*: Kyowa Kirin, Recordati Rare Diseases, Novartis. Rafael F. Duarte: − Honoraria*: Gilead, Jazz, MSD, Omeros, Sanofi, Sobi, Takeda. − *Ownership interests:* OrcaBio. Francine Foss: − *Consultancy/Advisory Board*: Secura Pharma, Kyowa Kirin, Citius, Acrotech. − *Investigator*: Kyowa Kirin, Electra, Ono Pharma, Dren Bio. Eva González‐Barca: − Honoraria*: Johnson&Johnson, AbbVie, Kyowa Kirin, Incyte, Astra Zeneca, Takeda, Roche. − *Consultancy/Advisory Board*: Johnson&Johnson, AbbVie, Kyowa Kirin, Incyte, Lilly, BeOne, Roche. − *Travel support*: Johnson&Johnson, AbbVie, Astra Zeneca, BeOne, Roche. Joan Guitart: Nothing to disclose. Pablo Ortiz‐Romero: − Honoraria*: Kyowa Kirin, Recordati Rare Diseases, Ideogen. − *Consultancy/Advisory Board*: Kyowa Kirin, Ideogen, HAC Pharma, Takeda. − *Travel support*: Kyowa Kirin, Almirall, Novartis, Takeda. Alessandro Pileri: − Honoraria*: Kyowa Kirin, Recordati Rare Diseases, Takeda. − *Consultancy/Advisory Board*: Kyowa Kirin, Recordati Rare Diseases Takeda. Pierluigi Porcu: − *Consultancy/Advisory Board*: Innate Pharma, Kyowa Kirin, Lilly‐LOXO, Artiva (Data Safety Monitoring Board). − *Research funding/Grants*: Innate Pharma. Henry M. Prince: − Honoraria*: Kyowa Kirin, Takeda, Mallinckrodt. − *Consultancy/Advisory Board*: Takeda, Kyowa Kirin. − *Research funding/Grants*: AbbVie. − *Travel support*: Prescient. Sima Rozati: − *Consultancy*: KKI, Therakos, SecuraBio, Helsinn. − *Research funding/Grants*: KKI. Serena Rupoli: − Honoraria*: Kyowa Kirin, Takeda. − *Consultancy/Advisory Board*: Kyowa Kirin, Takeda. − *Travel support*: Kyowa Kirin, Takeda. Maarten H. Vermeer: − Honoraria*: Kyowa Kirin, Recordati. − *Research funding/Grants*: Kyowa Kirin, Recordati, Immios. Sean Whittaker: Nothing to disclose. Nicola Pimpinelli: − Honoraria*: Kyowa Kirin, Recordati Rare Diseases, Takeda. − *Consultancy/Advisory Board*: Kyowa Kirin, Recordati Rare Diseases, Takeda. Pier Luigi Zinzani: − Honoraria*: Recordati, Kite Gilead, Janssen‐Cilag, BMS, SOBI, MSD, Regeneron, Takeda, Roche, AstraZeneca, Kyowa Kirin, Novartis, Incyte, BeOne Med., Eli Lilly. − *Consultancy/Advisory Board*: Secura Bio, Recordati, Kite Gilead, Janssen‐Cilag, BMS, SOBI, Sandoz, MSD, Regeneron, Takeda, Roche, AstraZeneca, Kyowa Kirin, Novartis, ADC Therap., Incyte, BeOne Med., Eli Lilly. *Honoraria (lectures, speaker bureaus, educational activities).

## FUNDING

This research received no funding. Open access publishing facilitated by Universita degli Studi di Milano, as part of the Wiley ‐ CRUI‐CARE agreement.

## Data Availability

Data sharing is not applicable to this article as no data sets were generated or analyzed during the current study.
